# Optofluidics in Microstructured Optical Fibers

**DOI:** 10.3390/mi9040145

**Published:** 2018-03-24

**Authors:** Liyang Shao, Zhengyong Liu, Jie Hu, Dinusha Gunawardena, Hwa-Yaw Tam

**Affiliations:** 1 Department of Electrical and Electronic Engineering, Southern University of Science and Technology, Shenzhen 518055, China; shaoly@sustc.edu.cn (L.S.); s1604080303@stu.cjlu.edu.cn (J.H.); 2 Photonics Research Center, Department of Electrical Engineering, The Hong Kong Polytechnic University, Kowloon, Hong Kong; dinusha.gunawardena@polyu.edu.hk (D.G.); eehytam@polyu.edu.hk (H.-Y.T.)

**Keywords:** optofluidics, microstructured optical fiber, MOF, sensors

## Abstract

In this paper, we review the development and applications of optofluidics investigated based on the platform of microstructured optical fibers (MOFs) that have miniature air channels along the light propagating direction. The flexibility of the customizable air channels of MOFs provides enough space to implement light-matter interaction, as fluids and light can be guided simultaneously along a single strand of fiber. Different techniques employed to achieve the fluidic inlet/outlet as well as different applications for biochemical analysis are presented. This kind of miniature platform based on MOFs is easy to fabricate, free of lithography, and only needs a tiny volume of the sample. Compared to optofluidics on the chip, no additional waveguide is necessary to guide the light since the core is already designed in MOFs. The measurements of flow rate, refractive index of the filled fluids, and chemical reactions can be carried out based on this platform. Furthermore, it can also demonstrate some physical phenomena. Such devices show good potential and prospects for applications in bio-detection as well as material analysis.

## 1. Introduction

Optofluidics has become more and more attractive and promising in the recent decade due to its features of small consumption of volume, fast response, and high sensitivity, as well as its capability of interacting light with substance, especially with chemical solutions [1]. Basically, it combines the disciplines of microfluidics and waveguides so that light-matter interaction can occur in direct or indirect ways. Typically, direct interaction means that the liquid flows in the channel also act as the optical core (e.g., hollow-core waveguide [2,3]), whereas indirect interaction means that liquids and light are separated but mutually influenced via indirect means (e.g., evanescent field [4], surface plasmonic resonance [5], heat flux [6], mechanic force [7]). Many interesting applications of optofluidic devices have been investigated, such as lab-on-chip and lab-in-fiber devices. Through the use of such technology, it is possible to easily achieve live cell imaging, chemical synthesis, particle trapping/sorting, biological analysis, high performance sensors (for e.g., DNA, refractive index (RI), flow rate), etc. [8]. However, most of these applications are carried out on a chip with customized microchannels. These channels vary from tens to hundreds of micrometers to allow for different analytes or particles in solution to pass through and eventually be analyzed. Microfluidics has blossomed extensively thanks to lab-on-chip technology [9]. To fabricate the tiny microchannels on a chip made of, e.g., SU-8, PDMS, silica, etc., micro/nanofabrication technologies such as photo- or soft-lithography are necessary, which, to some extent, increases the fabrication complexity and cost. Nevertheless, microfluidics provides a good platform for biological and chemical analysis, especially after being integrated with optics, which has occurred over the past 10 years.

In addition to lab-on-chip technology being an excellent platform to conduct microfluidics due to the flexibility of designing microchannels, specialty optical fiber is another promising option to combine microfluidics with optics owing to the light guidance in the fiber, which is thus called lab-in-fiber [10]. By microfabricating structures on the fiber facet where the light meets measurand, the refractive index of a specific liquid can be easily characterized using the hybrid metallo-dielectric nanoprobes [11] or Fabry–Pérot (FP) cavity [12]. As reported recently in 2017, a microbubble was also fabricated on the fiber end facet to realize an optofluidic interferometer [13]. Apart from the microstructures on the fiber tip interacting with liquids, the gap between two right-cleaved optical fibers is employed to conduct optofluidic measurement as well. Typically, such a gap is part of the microchannel where the target liquid flows. Two fiber Bragg gratings (FBGs) inscribed on these two fibers form an FP cavity that covers the gap so that the change of the fluids (e.g., RI, flow rate) can be detected by the optical resonance signal [14,15]. Furthermore, Leite et al. reported in 2017 that the light coming out from the end facet of a high NA (numerical aperture ) multimode fiber can trap the particle tightly and move it in three dimensions [16], which provides the technology to manipulate particles or cells in optofluidics.

Although the fiber end facet is a good place for light to come out and interact with fluids directly, the contact area is still limited. The advent of microstructured optical fibers (MOFs), especially hollow-core photonic crystal fiber (PCF), offers a perfect platform for guiding the fluids and light on the same path and along a longer length to make direct/indirect interaction possible. What is more, MCF fibers made of silica are more resistant to external interference compared to plastic microchips due to the properties of silica, such as being inert, having low auto-fluorescence, being non-deformable under high pressure, and so on. Microstructured fiber can also be suitable for remote sensing because of the fact that MCF can be fabricated in kilometer lengths at low cost. The air hole region acts as a place for chemical reaction under the core light irradiation, which is very promising in the applications of chemical sensing and photochemistry [17]. Using such a technique, the process of catalytic reaction could be analyzed in ultralow concentration due to the tiny amount of sample filled in the core [18], which, in contrast, is not easy to achieve in a conventional photoreactor system. Under the irradiation of the light guided in the hollow core, the conversion from cyanocobalamin (CNCbl) to aquacobalamin (H_2_OCbl) was demonstrated by Unterkofler et al., which is comparable to the reaction in the cuvette. Moreover, one study reported that the qualitative detection of Cy-5-labeled DNA molecules in aqueous solution is made possible by filling a PCF with the sample and measuring the transmission absorption [19]. Additionally, a good review on the development of optofluidic PCF-based sensors was recently put forth by Ertman et al., in which RI, mechanical quantities, electrical field, and magnetic field sensors employing optofluidics in PCFs are reviewed [20]. Overall, interest has increased significantly in the applications of optofluidics in MOFs.

In this paper, we give a short review of the optofluidics based on the platform of MOFs, especially focusing on the techniques used to implement optofluidic sensors and their applications. In next section, a brief introduction of MOFs together with the technical issues of inlet/outlet are given, followed by the encouraging applications realized by MOF optofluidics. The conclusion is presented at the end.

## 2. Implementation of Optofluidics Based on Microstructured Optical Fibers

**Figure 1:**
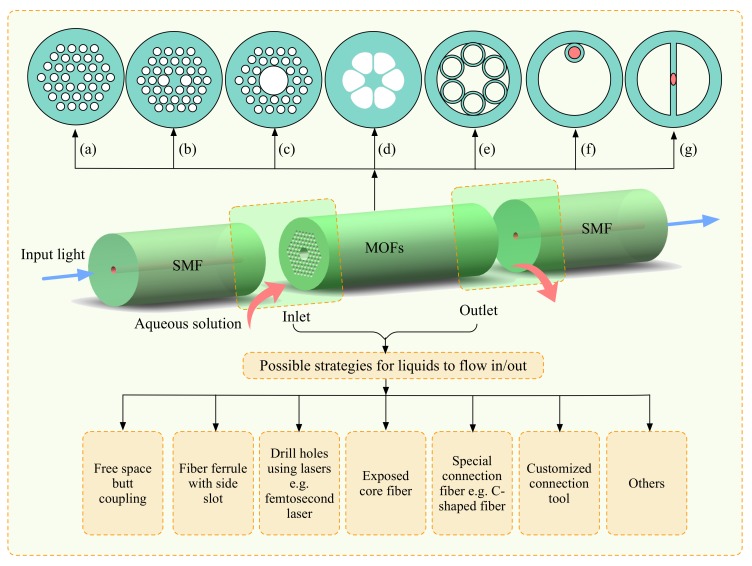
Schematic figure of the optofluidics based on various microstructured optical fibers (MOFs).

Since the first PCF was invented and fabricated by Phillip Russel et al. in the 1990s, they have attracted increasing attention from researchers in various disciplines [21]. Many fiber devices based on PCFs have been developed. MOF is such a specialty optical fiber that possesses air holes arranged in a certain structure along the fiber length, and PCF is a type of MOF that consists of a honeycomb structure with air holes compared to silica glass, as shown in Figure 1a–c. It is worth noting that the structure of MOFs can also be realized by using lower-index rods instead of air holes; however, this structure is not suitable for the use of optofluidics as no air channels exist in the fiber. To achieve optofluidics where microchannels are needed, fibers with an air hole structure are desirable. MOFs are more general and have more flexibility in fabrication as long as the structure is able to confine light. The structure of an MOF is not limited to the periodic structure arrangement, which makes it possible to maintain a few large air holes in the cladding [22,23] while suspending a small core in the center, as shown in Figure 1d–g. Therefore, these MOFs can be considered as a good option when employing evanescent field as an indirect interaction in optofluidics. In particular, there are two types of MOFs. One consists of a solid core, whereas the other consists of a hollow core. Typically, the former follows the light-guiding mechanism of total internal reflection similar to conventional optical fibers, whereas the latter MOFs obey the theories of either photonic bandgap (PBG) [24] or anti-resonant reflecting optical waveguide (ARROW) [25].

Particularly, the single-mode fiber consists of a core and cladding, which have a high and relatively low refractive index, respectively, and the light is guided based on total internal reflection (TIR). The MOFs with a solid core follow a similar guiding mechanism, in which the average index of the cladding is lower than that of the solid core due to the existing air holes. Therefore, it can also be called a modified TIR (M-TIR), and the MOFs obeying this principle can be called index-guiding MOFs. In terms of the hollow-core MOFs, the core is completely air, and its index close to 1, much lower than that of glass. In this case, TIR is not satisfied. However, owing to the periodic structure consisting of a low index part (e.g., air holes) and a high index part (e.g., glass), it has a Bragg reflection effect, which leads to several photonic bandgaps that allow light to be confined in the core. The certain wavelengths of the light leaking into the cladding can be reflected back into the core. Basically, the liquids filled in the hollow core have lower index than glass, thus the photonic bandgap should be applied as well to demonstrate optofluidics in this kind of MOF.

The index-guiding MOFs have air holes arranged in the cladding, which can be fully or selectively filled with an aqueous solution. When the core is reduced to 1–2 µm, the evanescent field is strong enough to penetrate into the air holes and interact with the fluids filled inside. On the other hand, the average refractive index of the air holes region changes with the filled fluids, which in turn influences the propagation constant of the core mode. For example, the RI sensitivity can be up to 38,000 nm/RIU (refractive index unit) when selective air holes are filled with liquid, resulting in a detection limit of 4.6 × 10^−7^ [26]. However, the light-matter interaction is more straightforward for the hollow-core MOFs as the light and fluids propagate in the core simultaneously. Most PCF-based optofluidics for photochemistry use hollow-core MOFs [17]. A liquid-core fiber proposes another interesting application, where the specific fluids infiltrated in the center hole become a new optical core and the optical properties of these liquids (e.g., nonlinearity) are employed to achieve photonic phenomena such as hybrid solitons and supercontinuum generation [27]. Hence, the flexibility of design as well as the fabrication makes MOFs very promising and attractive for optofluidics.

Figure 1 summarizes the state-of-the-art implementations of optofluidics based on various MOFs and the possible strategies that have been adopted to fabricate the inlet/outlet for the aqueous solution. The air holes in the cladding of the PCFs can be fully [28] or partially [29] filled with liquids to realize high sensitive RI sensors. Basically, the liquids with low viscosity can flow into the air channels automatically due to the effect of capillary force. However, an additional pump may be needed for highly viscous fluids [3]. Index-guiding PCFs as well as hollow-core PCFs are two main fibers used to conduct optofluidics, and the latter has been demonstrated to function excellently in photochemistry. In 2017, Gao et al. successfully demonstrated that in an anti-resonant MOF (e.g., Figure 1e), where light is guided based on the principle of ARROW, the RI and flow rate can be measured simultaneously [30]. This provides a good paradigm to carry out optofluidics in such a type of fiber. Moreover, the flexibility of fabricating MOFs allows the existence of large air holes, as shown in Figure 1, which makes it easier for liquids to enter the fiber. The tiny core of the fiber can be suspended in the center by three, four, or six large air holes [4], as shown in Figure 1d, or attached to the inner surface of a capillary [31], as shown in Figure 1f. The light-matter interaction of such fibers with large holes occurs due to the strong evanescent field from the tiny core.

To achieve optofluidics in MOFs, regardless of which type of fiber is employed, it is technically challenging to fill the aqueous solution into the air holes of MOFs properly, especially for solutions with high viscosity. There have been some excellent techniques reported to enable liquids to flow into the air holes of MOFs [2,4,15,17,28,29,32,33,34]. The initial and easy way is to butt-couple light into the MOFs, leaving a gap between the SMF (single mode fiber) and MOFs so that fluids can enter the air holes [2]. However, this requires very careful alignment and additional precise XYZ stages. Alternatively, the idea of inserting two fibers into the ferrule with a side slot has been realized, where the fluids enter via the open slot [35]. The free-spacing coupling has been found to be a convenient strategy because no additional special fibers or structures are needed. Another straightforward way is to directly drill entrance holes in the outer surface of MOFs, which can open specific inner holes of MOFs to external access [30]. In this way, the MOFs can be spliced to SMFs easily in advance; however, this method relies on high power laser technology such as femtosecond laser micromachining. In terms of the suspended-core MOFs, T.M. Monro et al. pioneered the utilization of exposed-core fiber to conduct optofluidics, where one of the three holes in the fiber is mechanically polished and opened during the preparation of the fiber [4]. Eventually the tiny fiber core is exposed and can interact with and sense the materials based on fluorescence. In 2013, Wu et al. adopted a brilliant way of connecting a short C-shaped fiber between an SMF and PCF to measure the RI, where the water can enter the air holes of the PCF via the open C-shaped fiber [28]. This technology allows the MOF-based optofluidic device to be assembled using only a procedure of splicing. In addition to these special fibers, other customized connection tools have been developed as well to provide entrance for the fluids, including microfluidic chips capable of mounting fibers [3] and capillary-assisted arrangements [34]. Similar to the lab-on-chip setup, a syringe pump can be utilized for all of these strategies to improve the filling efficiency, especially when the fluids have relatively high viscosity.

The flexibility of MOFs fabrication as well as the inlet/outlet design makes the optofluidics implemented in MOFs quite promising and encouraging. No additional waveguide for light is needed. Since all of the test configurations are fiber-based, such optofluidic devices could be very miniature. The mature fiber-optic sensing technologies, e.g., grating techniques (i.e., fiber Bragg grating (FBG), long period grating (LPG), tilted fiber Bragg grating (TFBG), etc.), as well as interferometry (i.e., Fabry–Pérot interferometry, Sagnac interferometry, Mach-Zehnder interferometry, etc.), can be used. Moreover, fluoroscopy and the absorption of materials have been widely utilized to sense analytes filled in MOFs. It can be anticipated that, together with lab-on-chip microfluidics, the platform of MOFs will play an increasingly important role in optofluidics.

## 3. Applications

### 3.1. Microfluidic Sensing Mechanism

According to the classification of sensing mechanisms, like other optical fiber sensors, microstructured optical fiber sensors can be broadly divided into light intensity absorption-based sensors, wavelength-shift-based sensors, Stimulated Raman-scattering-based sensors, and fluorescence-based sensors.

**Figure 2:**
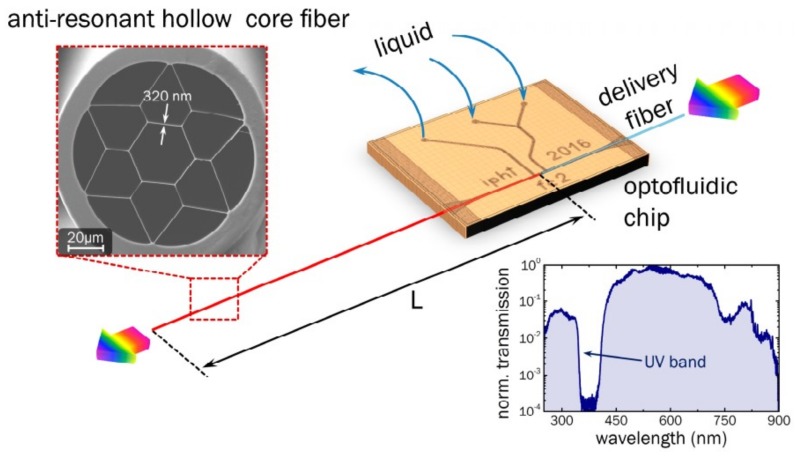
Schematic of the sensor for pharmaceuticals based on UV spectroscopy and water-filled anti-resonant hollow-core fibers (red), as well as the microfluidic chip (orange) and the delivery fiber (light blue), which are proposed in Reference [36]. The two insets show a scanning electron micrograph image of the cross-section of the silica microstructured fiber (upper left, the number refers to the average strand thickness in nm) and an example sample transmission spectrum (normalized to the maximum transmission value at 560 nm) where the fiber is filled with water (lower right).

Light intensity absorption-based detection is one of the most commonly used detection methods. It is well known that the evanescent energy distributed in the microfluidic channel of the microstructured fiber would be absorbed by the analyte, which influences the intensity of the transmitted light. This sensing mechanism is typical of fiber-based chemical sensors and traditional analytical instruments. Although different wavelengths can be chosen, UV-Vis-NIR absorption is one of the most commonly used light-based methods for the chemical identification of analytes because many analytes can be detected by their absorption changes in this region. For instance, Mona Nissen et al. introduced the concept of UV spectroscopy in liquid-filled anti-resonant hollow-core fibers to carry out the detection of pharmaceuticals, as shown in Figure 2. The limit of detection (LOD) for sulfamethoxazole (SMX) and sodium salicylate (SS) reached down to 0.1 µM (26 ppb) and 0.4 µM (64 ppb), respectively [36].

It is already a well-known mechanism to detect the shift of the transmission spectrum due to the refractive index variation of the analyte. Despite the relatively good sensitivity, there are many interesting ways to further improve its performance. Surface plasmon resonance (SPR) is the most well-known method. A wavelength-shift-based sensor is linked to refractive index sensing, and will be covered in more detail in the next section.

Raman spectroscopy is a powerful tool for determining molecular structure and has been widely used in many aspects. For example, V. S. Tiwari et al. monitored the concentration ratio of liquid oxygen and nitrogen in a cryogenic mixture based on a fiber-optic Raman sensor [37]. However, the Raman scattering is extremely weak, and thus a high-sensitivity spectrometer and a high-power pump source are necessary. Obtaining an adequate Raman signal of analyte molecules in an optical fiber has become a hot topic [38,39]. F. M. Cox et al. first demonstrated that surface-enhanced Raman scattering (SERS) signals can be enhanced by the guidance in a hollow-core MOF [40]. A. Amezcua-Correa et al. proposed a SERS probe based on an MOF substrate, where the metal nanoparticles were deposited on the inner surface of the air channels of the MOF [41]. The next year, H. Yan et al. designed surface-enhanced Raman probe based on an index-guided PCF, and compared the performance of SERS obtained when the nanoparticles were coated on the inner surface of the air holes and mixed in the analyte solution. The experimental results show that by mixing the gold nanoparticles in the solution, the sensor can attach more analyte molecules, thus leading to a higher sensitivity [42]. G. Wang et al. explored gas Raman sensing in a multi-opened-up suspended-core fiber, where the multi-opened-up structure accelerated the gases diffusion. As a result, real-time Raman sensing could be realized with a response time of less than 6 s [43].

Fluorescence is the photoluminescence of cold luminescence, i.e., the light of a longer wavelength emitted by a substance that absorbs light or other electromagnetic radiation, which has been widely used in microscopy, environmental pollutant monitoring, and biosensing for many years. Some scholars use optical fibers with a tiny core to fabricate sensors, so that there is a large amount of evanescent field energy interacting with the analyte, leading to light absorption or a fluorescence effect that can be utilized for sensing. Optofluidics in MOFs integrate the light path and fluid together into a single strand of fiber so as to increase the interaction length between the light field and analyte, which eventually can improve the sensitivity of fluorescence-based detection [44,45]. S. C. Warren-Smith et al. presented two new methods to fabricate exposed-core fiber and demonstrated that, compared with traditional MOFs with a protected core [4], such exposed-core MOFs can achieve a shorter response time in fluorescence detection. It is worth noting that not all substances can produce fluorescence. In addition to the fact that those limited types of substances that produce fluorescence are often used as fluorescent markers to detect other materials, fluorescence detection is often associated with biochemical sensing or other molecular detection [46,47].

### 3.2. Microfluidic Detection Applications

#### 3.2.1. Physical Parameters Detection inside Fluids

##### Microfluidic Flow Rate Detection

**Figure 3:**
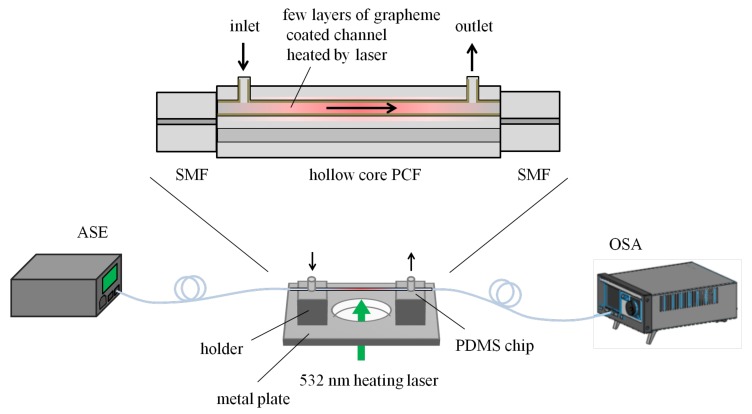
The schematic of the experimental setup for flow rate detection in Reference [30].

The microfluidic flow rate is a critical parameter in the industrial, chemical, and biological realms. Precise control of the flow rate has been widely used in nanotechnology and cell biology. Traditional flow rate optical fiber sensors are mostly based on the lateral force of a fluid acting on the surface of the fiber probe, making the fiber optic probe bend, causing the strength or phase of the optical signal transmitted within the fiber to change, and thus achieving the purpose of detection [48,49]. With the popularity of lab-on-chip designs, many teams have integrated fiber sensing probes with polymers into a single chip [7,50]. The microfluidic channel for liquid flow crosses the fiber channel. The chip integration not only reduces the size of the sensor but also reduces the amount of sample required. Microstructured fibers combine the optical path with the liquid flow path inside the fiber, which greatly increases the sensor’s integration. Such fluid flow in this way will not bend the fiber. However, fluid flow can take away heat. P. Christodoulides’s research group explored the relationship between liquid flow and heat transfer in the fiber [51]. Zhimei Qi’s research group proposed an anti-resonant reflecting guidance, a hollow-core photonic crystal fiber coated with a few layers of grapheme on the surface, and realized the simultaneous measurement of flow rate and refractive index [30]. The graphene film grown using the chemical vapor deposition technique was rinsed in deionized water several times and floated on the water surface. The graphene was rinsed into the hollow-core photonic crystal fiber with water, and coated on the surface of the hollow-core after the water was dried. Figure 3 shows the experimental setup of this sensor. A 532-nm laser connected with a beam expander was used to illuminate the few layers of grapheme in the hollow-core PCF through the bottom hollow slot of the metal plate. As the fluid flows at different velocities in the microfluidic channels in the fiber, the heat transfer rates and refractive index of the grapheme layers are also different, resulting in different transmission intensities. The experimental results show that a sensitivity of up to −2.99 dB/(µL/s) was achieved, and could be further improved by increasing the heating intensity.

##### Refractive Index Measurement

Refractive index is a basic attribute of matter, indicating the ratio of the velocity of light in vacuum to the speed in this optical medium. Refractive index sensing is always an interesting application, and there is no exception within the optofluidics in microstructured optical fibers.

G. Wang et al. incorporated a side-opened fluidic microchannel and suspended-core structure into a dual-core PCF, and explored RI detection based on the dual-beam interference mechanism. The phase shift from the change of the birefringence, which corresponds to the refractive index of the fluidic analyte, was calculated. As a result, the sensor showed a good sensitivity and the LOD reached 2.2 × 10^−6^ RIU [52].

The optical leaky loss is induced due to the slight difference of refractive index between the analyte and the optical fiber through the evanescent field interaction. G. Wang et al. designed a side-opened surface plasmon resonance (SPR) sensor based on a suspended-core fiber where the core is exposed and coated by an Au film. They detected the transmitted light intensity and the results show that the LOD was 2.3 × 10^−5^ RIU [53].

Because the change of RI of fluid analyte in the MOFs gives rise to the variation of optical path, most RI sensors detect the wavelength shift of characteristic spectral peaks or valleys with the variation of the RI.

G. Bertrand et al. designed a solid-core PCF-based SPR sensor. In this sensor, two large channels for liquid analyte in the fiber were plated with gold to excite the SPR mode and improve the sensitivity; the detection resolution could reach 7.2 × 10^−6^ RIU [54]. The RI sensor based on twin-core photonic bandgap fibers proposed by Y. Wu et al. could also reach a detection limit of 10^−6^ RIU at wavelength 1100 nm [55]. X. Yu et al. designed an RI sensor by writing a long-period grating in a PCF, and measured an RI ranging from 1.32 to 1.39. They obtained a sensitivity of 4.1 × 10^−6^ RIU [56]. K. C. Darran filled the liquid into selective air holes of a PCF, and found that the maximum sensitivity of this RI sensor could reach up to 38,000 nm/RIU, as well as achieve a detection limit of 4.6 × 10^−7^ RIU [26]. Multichannel sensing based on the Fano resonance of the whispering gallery modes in MOFs was also proposed with the sensitivity of tens of nm/RIU in TE (Transverse Electrical) mode or TM (Transverse Magnetic) mode [57]. Furthermore, the simultaneous measurements of refractive index and other parameters are demonstrated in References [30,58]. We summarized the detection performance of those sensors in Table 1.

#### 3.2.2. Chemosensing

##### PH Sensing

PH is a critical parameter of fluids, describing the degree of acid-base strength of aqueous solution, and the detection of pH is of great importance in industrial and chemical fields. F. M. Cox et al. drilled a lateral hole into one of the air channels of polymer optical fibers and used it for chemical sensing. Owing to the easy interaction between the side-opened core and the ambient analyte, this chemical sensor can realize real-time measurement. Bromothymol blue (BTB) was used as an indicator to test the sensing characteristics of a slotted microstructured polymer optical fiber, and the change in color of the BTB was used to show the change in the pH of the solution inside the fiber. The acidic solution and the basic solution corresponded to completely different spectra, although this was only a qualitative result. The experimental results demonstrated that the fiber can serve as a real-time evanescent wave absorption spectroscopy pH sensor using bromothymol blue as an indicator [59].

X. H. Yang et al. designed a pH sensor based on a polymer (PMMA) MOF, where the air channels were deposited by a cellulose acetate (CA) thin film doped with pH-sensitive fluorescence dye (eosin). Dynamic tests showed that the eosin-CA-MPOF (microstructured polymer optical fiber) probe can respond quickly, within 1 s, due to the nanoscale thickness of the membrane and its good hydrophilicity. Fluorescence intensity showed high stability at each pH value. The measured pH value ranged from pH 2.5 to 4.5, and could be further expanded from 1.5 to 4.5 if using surfactants hexadecyl trimethyl ammonium bromide [60].

##### Gas Monitoring

With the improvement of people’s awareness of environmental protection and the consequent strict standards for gas emissions, it is increasingly important to develop sensitive and efficient gas detection methods. Optofluidics in MOFs provide a potential method to develop a sensitive, real-time gas sensor with high integration. Experiments on PCF-based gas detectors were initially used with evanescent wave sensing in a PCF [61,62,63]. However, in this type of optical fiber sensor, the sensitivity obtained was very low due to the low overlap between the gas sample and the optical field energy and their weak interaction. In a pioneering experiment, T. Ritari et al. demonstrated gas (methane and ammonia) detection in a hollow-core PCF by observing the direct absorption spectra in the near-infrared band [64]. Using similar techniques, hollow-core PCFs have since been used in detecting acetylene (C_2_H_2_) [65] and ammonia (NH_3_) at 1.5 μm [66], and methane (CH4) at 3.3 μm [67].

For a few meters of fiber length, it may take several hours to fill by the free diffusion of gas in the air channels of the PCF. An important challenge for PCF gas detection is the ability to achieve a rapid response to sudden changes and real-time monitoring. It has recently been shown that lateral access holes can be made in the PCF cladding, allowing the gas to reach the hollow faster. Y. L. Hoo et al. cut out a 7-cm long piece of a hollow-core PCF, drilled seven mini-holes at the side of the PCF, and formed seven microchannels and the cross-section of a microchannel. A mixture of methane and nitrogen was inserted into the chamber of the hollow-core photonic bandgap fiber with a slow rate through these seven channels at the side, and the methane was detected with an LOD of ~647 ppm within a ~3-s response time [68]. S. H. Kassani et al. introduced a C-shaped fiber between multimode fibers and a suspended ring-core PCF, which made it easier for fluids to flow into the air channels of the PCF. In such a way, the sensitivity was improved and the response time was shortened [69]. X. Zhou et al. wrote a spiral trench on the surface of an SMF and wrote an FBG in the fiber, and then Pt-WO_3_ films, sensitive to hydrogen, were modified on the surface of the fiber. As a result, a hydrogen concentration from 0.02% to 4% at room temperature could be detected within a response time of 10~30 s [70].

##### Organic Chemicals and Solute Detection

**Figure 4:**
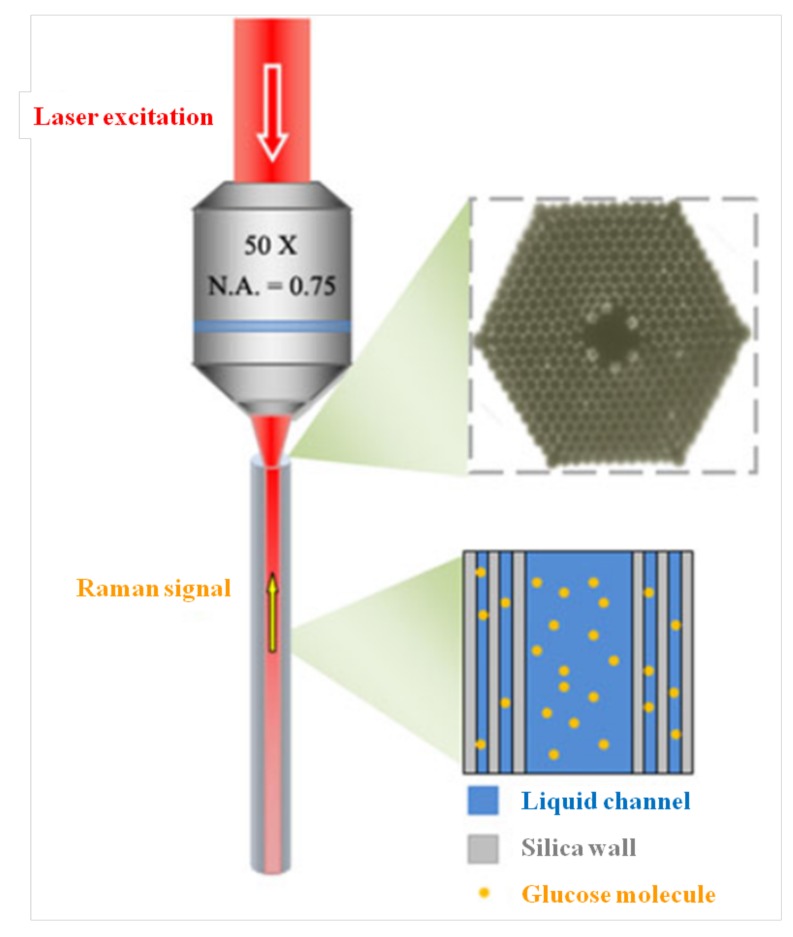
Schematic of a liquid-filled PCF probe for glucose detection proposed by Xuan Yang et al. Top right is cross-sectional view of the PCF probe. Bottom right depicts the air channels of the PCF filled with a glucose solution. Figure taken from Reference [74] with the permission of Springer Nature.

Due to the advantages of the technology and the detection platform, the use of PCFs for Raman spectroscopy is very common in chemical and biochemical sensing applications. Z. Xie et al. designed a broad spectral surface-enhanced Raman scattering (SERS) sensor by using a solid-core holey photonic crystal fiber with silver nanoparticles cluster. This SERS sensor was used to detect 4-Mercaptobenzoic acid. The strategy followed, contrary to previous works where high-pressure chemical deposition was employed, consisted of filling only 1 cm of fiber with Ag nanoparticles solution by capillary action. Compared to the signal obtained without Ag nanoparticles, higher intensities were obtained, as well as a good reproducibility of the results [71]. Yi Zhang et al. employed a hollow-core PCF to develop a fiber sensor based on SERS scattering. The hollow-core PCF was filled with liquid sample only in the core in order to maintain the bandgap and confine light in the hollow-filled region. More light energy interacting with the matter was achieved based on this method. The hollow-core PCF with a length of 10 cm was cut, and introduced to a 1-cm sample with silver nanoparticles (ranging from 40 to 60 nm in diameter) based on capillary action. Spectra of rhodamine 6 G, human insulin, and tryptophan were obtained by using the fiber as a Raman platform [72]. Not only Ag but also Au nanoparticles have been used to enhance the Raman signal. He Yan et al. proposed a SERS probe based on a hollow-core PCF in which Au nanoparticles were coated on the inner surface of air channels serving as a SERS substrate. A 5-cm long optical fiber was cut out and the Au nanoparticles (100–200 nm) colloidal was introduced (1–3 cm) by capillary action. After drying at 60 °C for 2 h, rhodamine B solution was introduced and dried. The signal of the analyte was verified after subtracting the background spectrum from the silica [73]. Xuan Yang et al. proposed a glucose biosensor based on a hollow-core PCF and Raman spectroscopy, as shown in Figure 4. An 8-cm long hollow-core PCF was used in the system, a liquid sample with d-glucose was introduced by capillary action, and then one end of the optical fiber was sealed. The peak signals from d-glucose measured in this system were in agreement with the direct analysis in a glass cuvette, while the intensity was 140-fold higher than the latter. Furthermore, they also prepared and measured solutions with different d-glucose concentrations, for which the LOD reached 1 mM [74].

As for fluorescence measurements, Cordeiro et al. detected rhodamine in solid-core MOF and hollow-core MOFs by selectively filling MOFs [75]. The sample introduction and optical alignment are independent because they do not need to be in contact with the fiber tip, which is very advantageous for optical sensing in a laboratory. Stanislav O. Konorov et al. tested various fused silica and soft glass PCFs to detect a dye (thiacarbocyanine) [76]. The large-diameter cladding air channels were filled with a liquid sample based on microcapillarity. Diode-laser radiation was delivered to a sample through the central core of the fiber, and the fiber cladding collected the fluorescent response from the dye and guided it to a detector. Cordeiro C. M. et al. designed a new method to selectively fill solid-core PCFs and hollow-core PCFs with a liquid sample [77]. The air channels were collapsed in one fiber end firstl, and then pressure was applied from another fiber end when the arc of a fusion splicer softening the PCF near the point where the side-hole was generated. Lateral filling of rhodamine was achieved for both structures, and the substance was successfully detected. Williams et al. employed a 30-cm length of water filling a hollow-core MOF to measure fluorescein in the magnitude of the attomole and achieved LOD of 0.02 nM [78].

In addition, absorption-based detection has been successfully achieved with PCFs. Martelli et al. proposed using a pure silica solid-core PCF to detect porphyrin in an aqueous solution based on UV-Vis absorption. After filling a 22-cm long solid-core PCF by capillary action with an aqueous solution containing the sample, which took 3 h, detection was performed by using an excitation laser at 640 nm and a optical spectrum analyzer [79]. X. Yu et al. demonstrated an evanescent field absorption sensing technique in liquid solutions using a microstructured photonic crystal fiber. A defected-core PCF made of silica was used to detect the cobalt chloride (CoCl_2_). This core consisted of a hole smaller than those from the cladding, and the evanescent field penetrated into the air channels, which enhanced the liquid absorption sensitivity. The air channels of the PCF were filled with the analyte solution by capillary action, following which concentrations of CoCl_2_ from 0.01 M to 0.5 M were detected with a good linear response [80].

#### 3.2.3. Biomedical Sensing inside Fluids

The air channels of MOFs serve as natural microfluidic channels, allowing MOFs to be potential biomedical sensors. Some researchers have demonstrated fiber-based biochemical sensors [81,82] through theoretical simulation, and explored the detection performance of biomolecules layer thickness. Md. Rabiul Hasan et al. numerically introduced surface plasmon resonance into the biosensor and further increased its sensitivity [83]. Zhengyong Li detected bovine serum albumin in experiments without specificity [84]. V. A. Popescu proposed a photonic fiber-based plasmonic sensor with a thin gold layer and 14 small air holes applied for the detection of human blood groups A, B, and O [85], which can also be applied for the determination of the hemoglobin concentration in normal human blood. Jian Sun et al. attempted to use goat antihuman IgG (antibody) for the specific detection of human IgG and yielded good results [86]. The sensitivity was found to reach around 0.1 nmol/L, and the required sample was less than 1 μL. Yi Zhang et al. employed a hollow-core PCF to develop a fiber sensor based on SERS scattering, and the spectra of human insulin and tryptophan were obtained [72].

**Figure 5:**
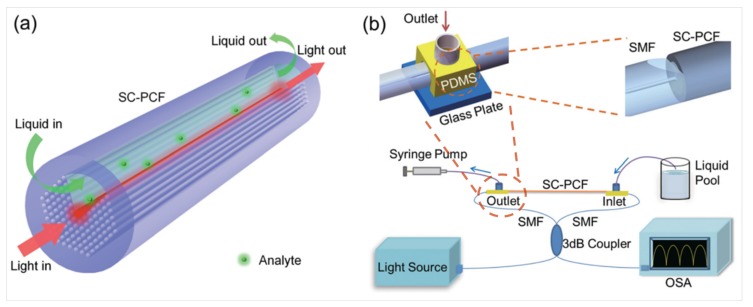
(**a**) Schematic diagram of liquid sensing with side-channel photonic crystal fiber (SC-PCF) (dimensions not to scale); (**b**) Details of the splicing point of the SC-PCF to side-polished single mode fibers (SMFs) and scheme of the absorption experiment; (Bottom) Scheme of the Sagnac interferometer. Figure taken from Reference [87] with the permission of the Royal Society of Chemistry.

**Figure 6:**
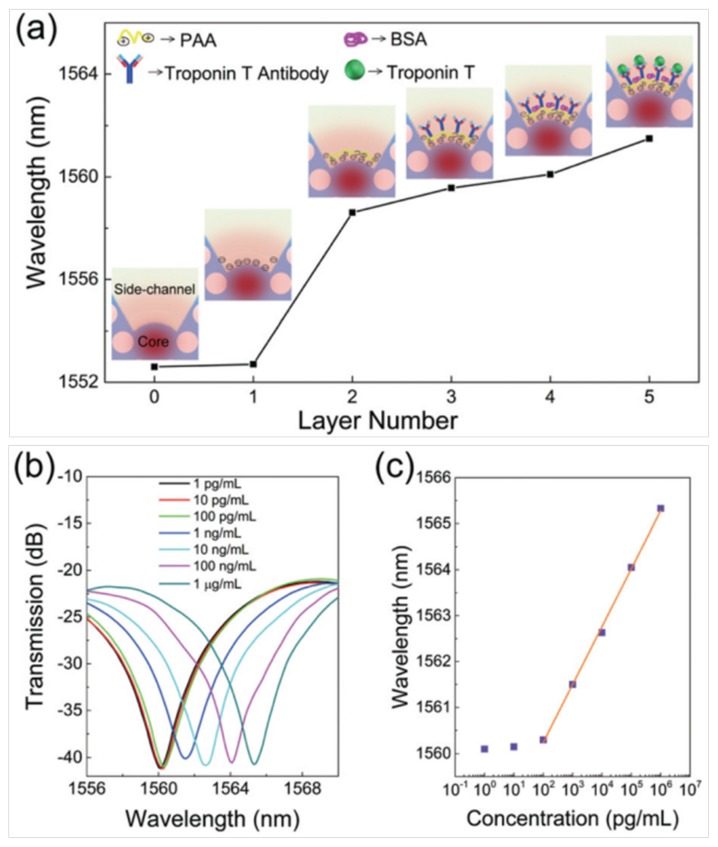
(**a**) Shift of the resonance wavelength monitored at various surface treatment steps (after rinsing thoroughly). The concentration of the human cardiac troponin T (cTnT) protein is 1 ng/mL. Insets are the illustrations of the binding profiles inside the side-channel. The red color region represents the mode profile in the fiber core and evanescent wave that extended to the side-channel. (**b**) Shift of the transmission spectra near the resonance wavelength of 1560 nm corresponding to the binding effect of different concentrations of the cTnT antigen. (**c**) Resonance wavelengths extracted from the spectra. The purple squares indicate experimental data and the orange straight line is linearly fitted to the experimental data. Figure taken from Reference [87] with the permission of the Royal Society of Chemistry.

Lei Wei’s research group designed and fabricated a biosensor based on a side-channel photonic crystal fiber (SC-PCF), which is shown in Figure 5a,b [87]. One-sixth of the cladding air holes was left blank, and a larger air channel was formed on the side of the core. As shown in the inset of Figure 5b, the fabricated SC-PCF was spliced with a side-polished SMF to simultaneously enable lateral liquid channeling and light transmission in the fiber core. In this system, the electrostatic interaction-based modification method was used to immobilize the human cardiac troponin T (cTnT) antibody on the fiber core surface, as shown in Figure 6a. The method of biomass immobilization based on electrostatic interaction is relatively convenient. The various surface treatment layers consist of hydroxide ions generated from sodium hydroxide solution, a monolayer of poly(allylamine) (PAA), and a layer of the active cTnT antibody. In addition, bovine serum albumin (BSA) was added to prevent non-specific binding. In the final step, a group of cTnT protein solutions (concentration ranging from 1 pg/mL onwards) was pumped into the side-channel to characterize its sensing capability and the LOD. We can see from Figure 6a that with the completion of each step of treatment, the spectrum produced a corresponding shift. Figure 6b,c show the spectra and resonance wavelength shift under different concentrations of cTnT antigen solutions. The final experimental results show that the LOD of this biosensing system reached 1 ng/mL.

**Figure 7:**
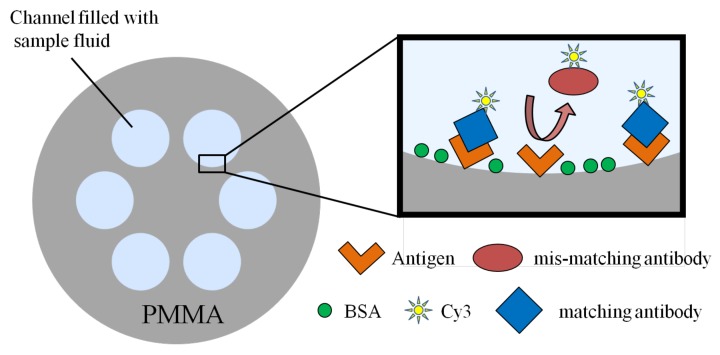
Schematic presentation of the capture processes utilized in the selective detection of the antibodies in Reference [88].

J. B. Jensen et al. immobilized the antigen (streptavidin molecules) inside the air holes of a polymer MOF and realized the selective binding of fluorophore-labeled antibodies (α-streptavidin-Cy3) with antigen, while α-CRP-Cy3 molecules which could not be bound with streptavidin molecules were washed out [88]. If there were any antibodies in the analyte solution, the fluorophore could be detected so as to realize the selective detection of the target material. The corresponding specific detection principle is shown in Figure 7. In their work, the antigen was immobilized on the surface of PMMA directly, which is much more convenient than the treatment on the quartz fiber surface. This is because the streptavidin molecules can bind directly to the polymer surface while still being able to bind the antibody. Besides, the realization of the selective detection of α-streptavidin-Cy3 is based on selective binding between antigen and antibody, because of which the specific recognition is of high efficiency. The experimental results show that this biosensor had a detection limit of 80 nM with only a 27-μL sample volume.

**Figure 8:**
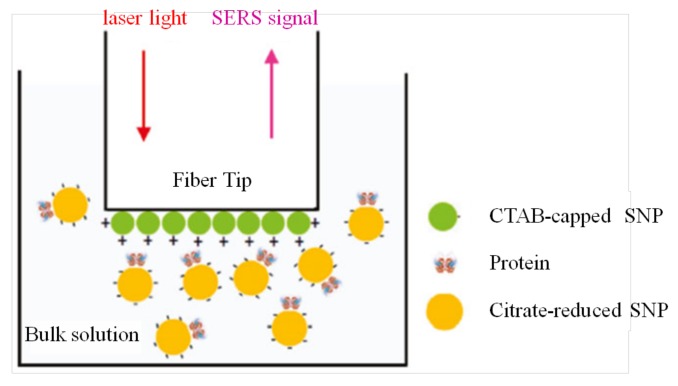
Scheme of the “sandwich” proposed by the authors for SERS sensing of proteins with an multi-mode fiber (MMF) (SERS: surface-enhanced Raman spectroscopy; CTAB: cetyltrimethylammonium bromide; SNP: silver nanoparticle). Reprinted (adapted) with permission from Reference [89]. Copyright (2011) American Chemical Society.

Xuan Yang et al. employed tip-coated multi-mode fibers (MMFs ) and a hollow-core PCF with SERS, and realized the detection of protein lysozyme and cytochrome C as well as live bacterial cells of Shewanella oneidensis MR-1 in an aqueous solution. Two systems were proposed. A tip-coated MMF was charged positively with Ag nanoparticles and proteins mixed with other negatively charged Ag nanoparticles were introduced, which then formed a sandwich structure as shown in Figure 8. The LOD of this system was as low as 0.2 mg/mL, an order of magnitude lower than the direct measurement using a glass cuvette. Another approach involved utilizing the a hollow-core PCF with the cladding sealed and confined light in the core, filled with the bacteria under study, obtaining LOD of 106 cells/mL [89]. Dinish U.S. et al. proposed a SERS biosensor for cancer proteins in low sample volumes based on a hollow-core PCF. Epidermal growth factor receptors, a common biomarker for various cancers, were immobilized on the walls of the core and Au nanoparticles were attached for SERS measurements. After protein immobilization by capillary action, which has an order of magnitude higher strength than traditional materials (glass slides), the SERS experiments were carried out. By introducing an extremely low volume, an amount of approximately 100 pg of cancer protein could be detected, which improved the sensitivity compared to other methods previously reported in the literature [90].

Another efficient method for the selective detection of a target material is based on molecularly imprinted polymer. A hybrid polymer containing target molecules was first produced on the surface of the optical fiber. Then the target molecules were separated from the polymer by hydrolysis, followed by rinsing with deionized water. As a consequence, some vacancies on the surface of the optical fiber were created, in which the target molecules can be embedded into exactly while other molecules are easily washed out. T. H. Nguyen et al. designed a fiber-optic biochemical sensor for the selective detection of cocaine based on molecularly imprinted polymer. The sensor detected an increasing fluorescence intensity in response to cocaine with an increasing concentration ranging from 0 to 500 μM in aqueous acetonitrile mixtures, and exhibited a good detection specificity compared with ketamine, ecgonine methyl ester, and other kinds of drugs [46]. Moreover, thanks to the stability of molecularly imprinted polymer, this kind of biosensor has good reproducibility. The biosensor reported in Reference [46] showed great selective detection performance even after one month.

**Figure 9:**
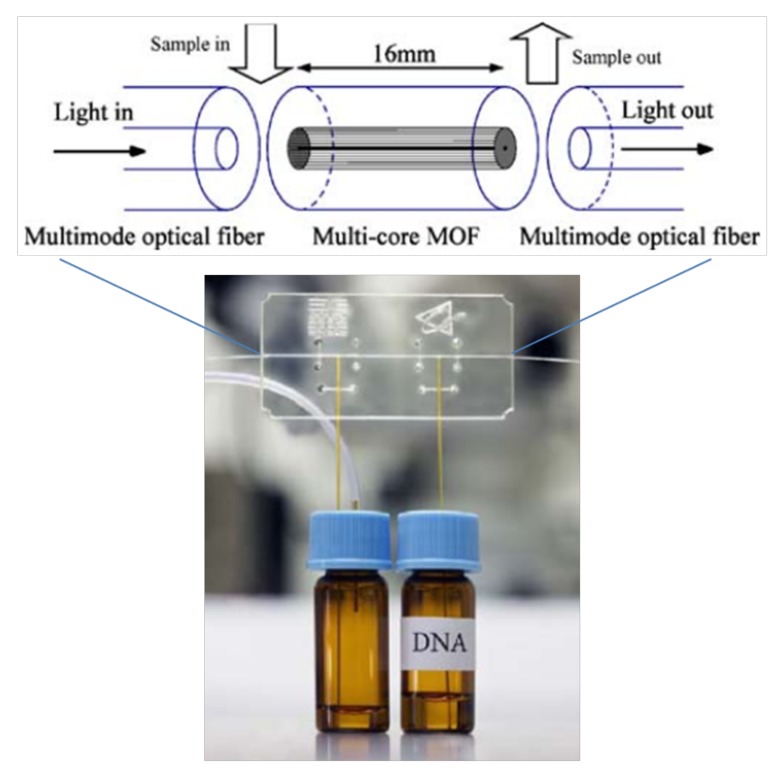
Scheme proposed by the authors where a lab-on-a-chip is integrated with an MOF, connected to two fibers to guide light. Fluid samples are injected through silica capillary tubes. Above, a scheme of the sensing area of the system is enlarged. Figure taken from Reference [91] with the permission of Springer.

From the biological protein concentration to the virus marker, it can be seen from the above investigation that the biosensor based on microstructured optical fiber has great potential. However, most of the current research remains in the theoretical simulation and preliminary experimental stages in the laboratory, and there is still a long way to go before mature practical application and productization. Lars Rindorf et al. have done a very good job in this area, as they presented the first incorporation of a microstructured optical fiber (MOF) into biochip applications, allowing the sample to flow continuously along the microstructure. Figure 9 shows a picture of this system and a scheme with the principal components in the sensing area of the system. A 16-mm long multi-core MOF of 125 μm outer diameter (OD), with a central air channels of 17 μm and a cladding composed of 312 channels of 2.3 μm, was integrated into a PMMA microchip. Theoretical calculations estimated that at the wavelength of 650 nm, around 6.5% of the field intensity, was in the air channels of the MOF. Techniques involving electrostatic coupling were employed to immobilize the DNA on the surface of the air channels of the MOF. After attaching, signals corresponding to the DNA were detected by comparing with the reference. Although this device had acceptable robustness, thermostatic control is required to improve its performance [91]. Yi Sun et al. took advantage of the exceptional heat-dissipation properties of PCFs and proposed to incorporate them into microcapillary electrophoresis chips. The PCF consists of a bundle of extremely narrow hollow channels, working as separation columns. A separation length of 7 cm was used in the PMMA microchip. After adding the dye (YOPRO-1) in the background electrolyte, DNA fragments could be detected by fluorescence measurements [92].

### 3.3. Other Applications

The magnetic nanoparticles solutions sensitive to an external magnetic field were infiltrated into PCFs. Harneet V. Thakur et al. reported a magnetic field sensor based on a polarization-maintaining PCF that infiltrated small amount of Fe_3_O_4_ magnetic optofluid/nanofluid in cladding holes, and a higher sensitivity of 242 pm/mT was realized [93]. Sully M. M. Quintero et al. proposed a magnetic field sensor comprised of a high birefringence photonic crystal fiber coated with a Terfenol-D/Epoxy composite layer, and the sensitivity of the developed sensor with magnetic fields was measured to be 6 pm/mT [94].

If the air channels of a PCF are filled with a substance that exhibits a strong electro-optical effect, particularly in liquid crystal that can effectively modify those optical properties through an electric field, the optofluidic PCF can also be used as an effective sensor for the electric field [95,96].

Super-continuous generation in the hollow-core PCF should also be mentioned, since it is one of the few PCF applications that has led to commercial products. Prathamesh S. Donvalkar et al. demonstrated high optical depths (50 ± 5) in Rubidium-filled hollow-core photonic band-gap fibers, which represented a 1000-fold improvement over operation times previously reported, in addition to lasting for hours. They also studied the vapor generation mechanism using continuous-wave and pulsed light sources and found that the mechanism that generates the Rubidium atoms is actually due to thermal evaporation [97].

## 4. Conclusions

In this paper, we reviewed the development and applications of optofluidics implemented based on MOFs, which have been demonstrated to be a promising platform with miniature size. The types of MOFs, especially those with hollow cores, are presented to emphasize the flexibility of designing air-hole structures for optofluidics. There are several main approaches to the fabrication of the inlet/outlet summarized in this study, which allow fluidics to enter the air holes of MOFs. In practical application, the approaches of drilling holes or using C-shaped fibers are more robust and reliable to achieve optofluidics. These approaches render it possible to make the entire system more compact compared with those using the butt-coupling method or other connection tools. Moreover, this technique is suitable for various kinds of MOFs, which then provides more flexibilities of configuring the optofluidic platform employing MOFs.

With a suitable design, particularly for biochemical applications, the MOF-based optofluidic platform can allow highly sensitive devices to sense fluidic flow rate, refractive index, and pH value, as well as conduct material analyses. Furthermore, it is also a potential tool to demonstrate physical phenomena with a small volume of materials. As a widely used technology, the fluorescence excited by specific substance filled in MOFs enables spectroscopic detection for biosensing. Among these, photochemistry is the most promising, especially when the system size and efficiency are critical concerns. Materials analysis could be one of the potential applications, as reviewed in this paper. The lab-in-fiber technology in MOFs provides the possibility of conducting chemical reactions only in the air channels of MOFs, which only requires a small volume.

For future prospects, the coupling of light and liquids into MOFs still raises research issues that attract attention. There are flexible air holes in MOFs, which means that these holes can be selectively filled with various liquids in order to conduct analyses. However, higher precision alignment and a better design of the inlet/outlet are required. Like the lab-on-chip technology, the microfluidic channels of an MOF can be designed and fabricated in other forms. In terms of good interaction between matter and light, the hollow-core MOFs and index-guiding MOFs with thin cores are better choices; however, their fabrication requires strict conditions and could be one future research topic aiming at the applications of optofluidics.

To conclude, optofluidics in MOFs open a whole new sector of useful applications for material characterization. The capability of propagating fluids and light simultaneously in MOFs makes them an excellent and encouraging platform for optofluidics.

## Figures and Tables

**Table 1:** Performance of refractive index (RI) sensors based on optofluidics in microstructured optical fibers.

Structure	Sensitivity	Limit of Detection	Reference/Year
Side-opened and suspended-dual-core fiber	8360 rad/RIU*	2.2 × 10^−6^ RIU	[52]/2012
Side-opened suspended-core fiber with surface plasmon resonance (SPR)	3500 nm/RIU	2.3 × 10^−5^ RIU	[53]/2015
Photonic bandgap fiber-with SPR	13,750 nm/RIU	7.2 × 10^−6^ RIU	[54]/2007
Twin-core all-solid photonic bandgap fibers		10^−6^ RIU	[55]/2009
Single-mode photonic crystal fiber (PCF) with long-period grating	243 nm/RIU	4.1 × 10^−6^ RIU	[56]/2008
Photonic crystal fiber with selective hole(s) filled by fluid	30,100 nm/RIU	4.6 × 10^−^^7^ RIU	[26]/2009
Exposed-core MOF with SPR	1900 nm/RIU~12,500 nm/RIU		[57]/2016
Graphene coated hollow-core PCF	1328 nm/RIU		[30]/2017
Capillary channels (different diameters) inside a tubular frame	13.3181 nm/RIU~29.0557 nm/RIU		[58]/2017

*RIU: refractive index unit.
